# Não Há Mais Tempo a Perder

**DOI:** 10.36660/abc.20240512

**Published:** 2024-09-12

**Authors:** Luiz Fernando Caneo

**Affiliations:** 1 Universidade de São Paulo Divisão de Cirurgia Cardiovascular São Paulo SP Brasil Universidade de São Paulo - Divisão de Cirurgia Cardiovascular, São Paulo, SP – Brasil

**Keywords:** Oxigenação por Membrana Extracorpórea, Choque Cardiogênico, Análise de Custo-Efetividade

A oxigenação por membrana extracorpórea (ECMO) é uma tecnologia avançada de suporte à vida que fornece suporte cardíaco e respiratório prolongado para pacientes cujos corações e pulmões não funcionam adequadamente. O conceito de utilização de uma máquina para substituir essas funções remonta a 1931, quando John Gibbon, residente cirúrgico em Boston, tratou uma mulher com embolia pulmonar.^1^ Sem os tratamentos disponíveis, ele só pôde vê-la deteriorar-se e falecer. Essa experiência levou Gibbon a imaginar um método para oxigenar o sangue fora do corpo em caso de obstrução vascular pulmonar. Em 1934, ele desenvolveu uma máquina que podia suportar a circulação de um gato durante trinta minutos e, em 1952, utilizou com sucesso a máquina em um ser humano, marcando o início da moderna cirurgia de coração aberto.^1^

Em 1971, Donald Hill^2^ utilizou um circuito extracorpóreo com um oxigenador especialmente projetado para dar suporte a um paciente com insuficiência respiratória durante trinta e seis horas, fazendo dele o primeiro ser humano a sobreviver com ECMO. Em 1975,^3^ Robert Bartlett e sua equipe utilizaram ECMO para salvar um recém-nascido com insuficiência respiratória. Nos anos seguintes, centenas de casos semelhantes foram tratados com uma taxa de sobrevivência de oitenta por cento, estabelecendo a ECMO como padrão nos principais centros pediátricos. O uso da ECMO em adultos se expandiu, principalmente para condições refratárias aos tratamentos convencionais durante a epidemia de H1N1 e posteriormente durante a pandemia de SARS-CoV-2.^4,5^ Esse aumento se deve às técnicas aprimoradas de canulação e aos avanços em bombas, oxigenadores e cânulas. Apesar destas melhorias, a seleção de candidatos adequados e a gestão dos seus cuidados diários continuam a ser um desafio.

Apesar de ser considerado padrão mundial de atendimento para suporte cardiopulmonar temporário em casos agudos refratários aos tratamentos convencionais, o Brasil só começou a utilizar a ECMO no início do século XXI.^6^ A primeira série clínica para pacientes cardíacos adultos e pediátricos foi publicada em 2008, seguida de casos respiratórios em 2012.^7-9^Atualmente, mais de 228.174 pacientes fazem parte do registro da Extracorporeal Life Support Organization (ELSO), mas apenas 2,5% desses casos foram realizados na América Latina (https://www.elso.org/registry.aspx). O número exato de casos de ECMO no Brasil é desconhecido, pois esta tecnologia, especialmente a ECMO respiratória, não foi incorporada ao sistema público de saúde e não está registrada como procedimento no banco de dados do Sistema Único de Saúde (SUS) (DATASUS).

Em 2014, Park publicou um estudo mostrando uma relação custo-utilidade potencialmente aceitável para o uso de ECMO em pacientes com síndrome do desconforto respiratório agudo grave no Brasil.^10^ Apoiada por uma revisão completa da literatura, incluindo este estudo, a proposta de incorporar à ECMO para suporte respiratório no sistema de saúde brasileiro foi submetida à Comissão Nacional de Incorporação de Tecnologia (CONITEC) em 2014 e novamente durante a pandemia. No entanto, ambas as submissões foram recomendadas para não incorporação.

Sem reembolso pelo SUS e cobertura limitada por seguros privados, a ECMO não tem sido amplamente adotada no Brasil, comprometendo a formação de recursos humanos e o desenvolvimento de centros especializados. A falta de recursos levou a resultados abaixo do ideal nos centros brasileiros, desencorajando ainda mais o uso generalizado da ECMO. A implementação da ECMO exige mais do que apenas ter o sistema disponível; necessita de equipes treinadas e hospitais especializados.

O choque cardiogênico (CC) permanece como um dos desafios mais complexos e alarmantes no tratamento cardiovascular agudo, marcado por sua variabilidade, crescente incidência e elevadas taxas de mortalidade.11 A oxigenação por membrana extracorpórea venoarterial (ECMO-VA) surge como uma estratégia promissora no tratamento do CC refratário, aumentando as chances de sobrevivência desses pacientes. A ECMO-VA desempenha um papel crucial no manejo da insuficiência cardíaca, restaurando e estabilizando a função dos órgãos.^12^ As indicações comuns para ECMO-VA incluem infarto agudo do miocárdio, miocardite fulminante, intoxicação por drogas cardiotóxicas, cardiomiopatia em estágio terminal, hipotermia, embolia pulmonar maciça e suporte pós-cirúrgico, incluindo pós-transplante. O suporte temporário de ECMO normalmente é necessário por alguns dias até que a função cardíaca se recupere.

No entanto, a adoção da ECMO-VA como suporte ao CC refratário em países de baixa e média renda como o Brasil levanta questões críticas sobre custo-benefício e acesso igualitário.

Um estudo recente realizado em centros terciários do sul do Brasil fornece informações essenciais, posicionando a ECMO-VA como uma terapia potencialmente custo-efetiva no SUS.^13^ Este estudo advoga a necessidade de realizar ensaios clínicos rigorosos, que abranjam perfis variados de pacientes, com o objetivo de confirmar a relação custo-efetividade e garantir o acesso justo a essas tecnologias que salvam vidas. Embora os argumentos econômicos a favor da ECMO-VA sejam convincentes, o estudo também destaca a questão premente da equidade nos cuidados de saúde. Garantir o acesso equitativo a intervenções médicas avançadas como a ECMO-VA é crucial num país marcado por disparidades sociais significativas. As conclusões do estudo abrem caminho para que formuladores de políticas e prestadores de cuidados de saúde considerem a ECMO-VA como uma opção padrão para CC refratário no âmbito do SUS.

É importante observar que o CC não se resume a uma questão mecânica ou hemodinâmica, mas envolve também fatores metabólicos complexos. Estratégias eficazes devem abordar ambos os aspectos para melhorar os resultados dos pacientes.^14^

Por enquanto, a ECMO-VA oferece uma chance real de sobrevivência para pacientes com choque cardiogênico refratário, sendo uma opção crucial na falha do tratamento convencional.

À medida que o Brasil luta pela igualdade nos cuidados de saúde, a integração de terapias avançadas como a ECMO-VA poderia representar um passo significativo para garantir que todos os pacientes, independentemente da sua condição socioeconômica, possam ter acesso aos melhores cuidados possíveis.
