# Supplementary material for: Não Há Mais Tempo a Perder
Source: Arq Bras Cardiol. 2024 Sep 12;121(8):e20240512. [Article in Portuguese] doi: 10.36660/abc.20240512 (PMC11495808; doi:10.36660/abc.20240512)
Supplement: Translation

# Translation: No Time to Die

**DOI:** 10.36660/abc.20240512i

**Authors:** Luiz Fernando Caneo

**Affiliations:** 1 São Paulo SP Brazil Universidade de São Paulo - Divisão de Cirurgia Cardiovascular, São Paulo, SP – Brazil

**Keywords:** Extracorporeal Membrane Oxygenation, Cardiogenic Shock, Cost-Effectiveness Analysis

Extracorporeal Membrane Oxygenation (ECMO) is an advanced life-support technology providing prolonged cardiac and respiratory support for patients whose hearts and lungs cannot function adequately. The concept of using a machine to replace these functions dates back to 1931 when John Gibbon, a surgical resident in Boston, treated a woman with a pulmonary embolism.^1^ Without available treatments, he could only watch her deteriorate and pass away. This experience led Gibbon to envision a method to oxygenate blood outside the body and bypass blockages. By 1934, he developed a machine that could support a cat's circulation for thirty minutes, and in 1952, he successfully used the machine on a human, marking the beginning of modern open-heart surgery.^1^

In 1971, Donald Hill^2^ used an extracorporeal circuit with a specially designed oxygenator to support a man with respiratory failure for thirty-six hours, making him the first human to survive on ECMO. In 1975,^3^ Robert Bartlett and his team used ECMO to save a newborn with respiratory failure. In the following years, hundreds of similar cases were treated with an eighty percent survival rate, establishing ECMO as a standard in major pediatric centers. The use of ECMO in adults has expanded, particularly for conditions refractory to conventional treatments during the H1N1 epidemic and later during the SARS-CoV-2 pandemic.^4,5^ This increase is due to improved cannulation techniques and advancements in pumps, oxygenators, and cannulas. Despite these improvements, selecting appropriate candidates and managing their daily care remains challenging.

Despite being considered a worldwide standard of care for temporary cardiopulmonary support in acute cases refractory to conventional treatments, Brazil only began using ECMO in the early 21st century.^6^ The first series of cardiac ECMO cases in adults and pediatrics were published in 2008, followed by respiratory cases in 2012.^7-9^ Currently, more than 228,174 patients are in the Extracorporeal Life Support Organization (ELSO) registry, but only 2.5% of these cases were performed in Latin America (https://www.elso.org/registry.aspx). The exact number of ECMO cases in Brazil is unknown, as this technology, especially respiratory ECMO, has not been incorporated into the public health system and is not registered as a procedure in the Unified Health System (SUS) database (DATASUS).

In 2014, Park published a study showing a potentially acceptable cost-utility ratio for using ECMO in patients with severe acute respiratory distress syndrome in Brazil.^10^ Supported by a thorough literature review, including this study, the proposal to incorporate ECMO for respiratory support into the Brazilian health system was submitted to the National Commission on Technology Incorporation (CONITEC) in 2014 and again during the pandemic. However, both submissions were recommended for non-incorporation.

Without reimbursement by SUS and limited coverage by private insurance, ECMO has not been widely adopted in Brazil, compromising human resource education and the development of specialized centers. The lack of resources has led to suboptimal outcomes in Brazilian centers, further discouraging the widespread use of ECMO. Implementing ECMO requires more than just having the system available; it necessitates trained teams and specialized hospitals.

Cardiogenic shock (CS) remains one of the most daunting challenges in acute cardiovascular care, characterized by its heterogeneity, increasing incidence, and high mortality rates.^11^ Venoarterial extracorporeal membrane oxygenation (VA-ECMO) has emerged as a beacon of hope, offering a lifeline to patients with refractory CS. VA-ECMO plays a crucial role in managing cardiac failure by restoring and stabilizing organ function.^12^ Common indications for VA-ECMO include acute myocardial infarction, fulminant myocarditis, cardiotoxic drug intoxication, end-stage cardiomyopathy, hypothermia, massive pulmonary embolism, and post-surgical support, including post-transplantation. Temporary ECMO support is typically needed for a few days until heart function recovers.

However, its adoption of VA-ECMO as a support refractory CS in low- and middle-income countries like Brazil raises critical questions about cost-effectiveness and equitable access.

A recent study in Southern Brazilian tertiary care centers provides pivotal insights, positioning VA-ECMO as a potentially cost-effective therapy within SUS.^13^ The study advocates for rigorous clinical trials encompassing diverse patient profiles to confirm cost-effectiveness and ensure fair access to these life-saving technologies. While the economic arguments for VA-ECMO are compelling, the study also highlights the pressing issue of healthcare equity. Ensuring equitable access to advanced medical interventions like VA-ECMO is crucial in a country marked by significant social disparities. The study's conclusions pave the way for policymakers and healthcare providers to consider VA-ECMO as a standard option for refractory CS within the SUS framework.

It is important to note that CS is not merely a mechanical or hemodynamic issue; it also involves complex metabolic factors. Effective strategies must address both aspects to improve patient outcomes.^14^

For now, VA-ECMO offers a chance for survival and serves as a beacon of hope for patients with refractory CS.

As Brazil strives for healthcare equity, integrating advanced therapies like VA-ECMO could mark a significant step toward ensuring that all patients, regardless of their socio-economic status, can access the best possible care.
